# The relationship between subjective social class and pro-social behavior: the mediating role of self-control and the dual-edged sword effect of shame

**DOI:** 10.3389/fpsyg.2025.1542045

**Published:** 2025-03-20

**Authors:** Jiaqi Zheng, Haoliang Liu, Zhifang He

**Affiliations:** ^1^School of Humanities, Jiangxi University of Chinese Medicine, Nanchang, China; ^2^School of Economics and Management, Harbin University of Science and Technology, Harbin, China

**Keywords:** subjective social class, pro-social behavior, self-control, shame, shame-proneness, state shame

## Abstract

**Introduction:**

Pro-social behavior is a widespread behavior in life that is beneficial to others and society. Previous research has focused on the influence of individual characteristics on pro-social behavior. The rise of social class psychology has provided a new perspective for the study of pro-social behavior. It has been shown that social class has an effect on pro-social behavior, but the mechanisms behind it have not been explored enough. This study explored in depth the mechanism of the influence of subjective social class on pro-social behavior.

**Methods:**

Study 1 examined the moderating effect of shame-proneness using a questionnaire with 312 subjects. Study 2 recruited 257 participants for an experimental study to examine the moderating effect of state shame.

**Results:**

(1) subjective social class positively predicted pro-social behavior. (2) Self-control partially mediated the relationship between subjective social class and pro-social behavior. (3) Both shame-proneness and state shame negatively moderated the relationship between self-control and pro-social behavior. These findings provided valuable insights for encouraging college students to be more prosocial, which is crucial for enhancing their moral standards and fostering a harmonious society.

**Conclusion:**

This study provides theoretical support for the causes and mechanisms behind the influence of subjective social class on pro-social behavior and has practical implications for the promotion of pro-social behavior.

## 1 Introduction

As a classic research area in psychology, the emergence and development of pro-social behavior (PSB) have long garnered the attention of psychologists worldwide (Liu et al., 2024). PSB encompasses actions and tendencies exhibited in social interactions, such as cooperation, sharing, comforting, and helping (Eisenberg et al., 2015). PSB is beneficial to the helper (Abel and Brown, 2022), and positively affects society’s development (Xu and Xie, 2023). Consequently, understanding the mechanisms underlying PSB promotion and identifying its causes remain central issues in psychological research (Yang et al., 2017).

Chinese’s ancient saying, “In success, one tries to let others be benefited,” seems to imply that individuals in higher social classes may be more prosocial. However, no definitive conclusions have been drawn regarding how social class influences an individual’s PSB (Zheng et al., 2023). Most early studies on this topic relied on samples from European and American countries, and their findings were relatively consistent within the Western social and cultural context. Specifically, individuals from lower social classes were found to exhibit higher levels of PSB (James and Sharpe, 2007; Piff and Robinson, 2017). Nonetheless, Miyamoto et al. (2018) pointed out that cultural differences may lead to variations in these patterns. Western cultures, which emphasize an independent self-construal, prioritize values such as personal agency, autonomy, and self-expression. In these cultures, individuals from higher social classes—who have greater access to resources and opportunities—tend to focus on personal goals and engage in activities that reinforce their independence (Kitayama et al., 2006). As a result, PSB may be less frequently observed among them (Piff et al., 2010), as their self-perception and socialization emphasize self-reliance over communal responsibility.

In contrast, East Asian cultures emphasize an interdependent self-construal, which values social harmony, collective well-being, and the maintenance of close interpersonal relationships (Triandis, 2018). Within this cultural framework, individuals from higher social classes often view their elevated status as a responsibility to improve the welfare of their social groups. The emphasis on relational obligations and moral duties in Confucian traditions further reinforces this tendency (Hofstede and Bond, 1988). Consequently, in Eastern cultural contexts, individuals in higher social classes are more likely to engage in PSB, which fosters social cohesion and mutual support (Ishida and Slater, 2009). The discrepancy between Western and Eastern findings underscores a gap in the literature: while the relationship between social class and PSB has been extensively examined in Western settings, its underlying mechanisms in Eastern cultural contexts remain insufficiently explored. Bridging this gap is essential for developing a comprehensive understanding of how cultural value systems shape the relationship between social class and PSB. Social class is comprised of objective social class (OSC) and subjective social class (SSC). Research has shown that an individual’ s SSC is more closely related to their psychology and behavior than their OSC (Kraus et al., 2013). Therefore, this article analyzed the relevant theoretical and empirical studies on SSC affecting PSB to clarify the relationship between them, helping people view the behavior of different social groups comprehensively and rationally, promoting mutual understanding between different class groups.

## 2 Literature reviews and hypotheses development

### 2.1 The relationship between of SSC and PSB

The relationship between SSC and PSB has been analyzed from multiple perspectives. One perspective, grounded in social cognitive theory, posits that lower-class individuals rely more on social support (Kraus et al., 2012), which heightens their sensitivity to others’ needs and increases their likelihood of engaging in PSB. Moreover, in highly stratified societies, lower class individuals often demonstrate a stronger willingness to help others (von Hermanni et al., 2019). However, such PSB is primarily driven by emotional resonance and practical necessity.

An alternative perspective suggests that high SSC individuals are more likely to engage in PSB due to more significant resources. The Cost Consumption Theory posits that implementing PSB requires resource expenditure (Korndörfer et al., 2015). Due to limited resources, lower-class individuals consume higher relative costs for engaging in PSB, thereby decreasing their likelihood of participation. Empirical studies support this perspective, demonstrating that high-SSC individuals are more actively engaged in philanthropy and volunteerism, leveraging their resources to advance social welfare (Stamos et al., 2020). Moreover, individuals from higher social classes typically possess broader social networks, which increase their susceptibility to social norms in interpersonal interactions, thereby facilitating prosocial behavior (Gradassi et al., 2024). While some studies suggest that lower-class individuals engage in PSB due to their dependence on social support (Piff et al., 2010), a broader body of literature indicates that high-SSC individuals, driven by resource advantages, are more predisposed to engage in PSB, hypothesis 1 is proposed: *SSC positively predicts PSB*.

### 2.2 The mediating role of self-control

While previous research has established a relationship between SSC and PSB, the psychological mechanisms underlying this association remain insufficiently explored. One potential explanation lies in self-control. Recent studies have highlighted the connection between social class and self-control, showing that adolescents from lower socioeconomic backgrounds consistently exhibit lower self-control (Jia, 2022). Robinson and Piff (2017) provided an explanation, arguing that individuals from lower social classes often struggle to maintain self-control due to the high levels of stress they experience, which in turn affects their health behaviors and social interactions. Beyond its association with social class, self-control is integral to fostering PSB. Li et al. (2022) demonstrated through a longitudinal study that enhanced self-control significantly promotes PSB in adolescents. However, their study primarily examined direct effects, overlooking variations across social classes. Notably, Self-control also functions as a key mediator in psychosocial relationships. Nie et al. (2016) found that it mediates the link between parental attachment and PSB, suggesting that higher self-control enables individuals to translate a positive family environment into increased PSB. Similarly, Zhong et al. (2024) identified self-control as a mediator between subjective socioeconomic status and environmentally responsible behavior, highlighting its broader influence on prosocial engagement, including sustainability efforts.

In summary, these findings suggest that socioeconomic status, family environment, and other contextual factors are closely related to an individual’s self-control capacity, which in turn affects their PSB (Suryadi et al., 2025). Building on these insights, Researchers propose that individuals with higher subjective social class tend to exhibit stronger self-control, which in turn increases their propensity to engage in PSB. Thus, this study formulates hypothesis 2: self-control mediates the relationship between SSC and PSB.

### 2.3 The moderating role of shame

Researchers have established that divergent perspectives exist regarding the relationship between SSC and PSB. This inconsistency in research findings indicates that some unexplored variables may influence the relationship. According to Hypothesis 2, self-control may play an essential role in SSC influencing PSB. From this perspective, if a certain variable can affect self-control resources, it may become a variable that affects the relationship between SSC and PSB. With the development of research related to self-control, researchers have begun to focus on the compensatory effects of moral emotions on self-control resources (Yang et al., 2023). Shame is a moral emotion that arises from an individual’s failure or moral anomie (Tangney et al., 2007). De Hooge et al. (2010) posits that shame can activate the motivation to restore the threatened self, thus generating approach behavior; on the other hand, when individuals believe that it cannot be restored, shame activates the motivation to protect the self, thus generating avoidance behavior. The Resource-Allocation Model of Self-Control points out that individuals regulate the allocation of self-control resources according to their behavioral motivations to achieve their goals (Beedie and Lane, 2012). Thus, in this framework, shame may moderate the role of self-control resources in supporting PSB by affecting individuals’ behavioral motivation. When shame stimulates restored motivation, individuals may actively mobilize more self-control resources to promote PSB. On the contrary, if shame mainly triggers avoidance motivation, individuals may fall into a state of depression and self-protection, and self-control resources are depleted, inhibiting the occurrence of PSB.

Shame can be classified into two categories. Shame-proneness is a long-term, stable tendency and emotional character, which is mainly measured by psychological tests; state shame is a transient feeling, which is primarily induced by experiments (Cohen et al., 2011). Trait and state emotions affect PSB differently (Haran, 2019). Leach and Cidam (2015) found that state shame promotes PSB. However, Ortiz Barón et al. (2018) used the self-awareness emotion test to find a negative correlation between shame-proneness and PSB. Ren et al. (2019) further found that state shame activates individuals’ motivation to restore their positive self, which increases individuals’ self-control resources, promoting more PSB (Yang et al., 2023). Therefore, we speculate that state shame (shame-proneness) activates the restoration (protection) motivation to regulate the effect of self-control resources on PSB. Based on this, a research hypothesis was proposed:

*H3:* The effect of self-control on PSB moderates by shame

*H3a (H3b):* The effect of self-control on PSB moderates by shame-proneness (state shame)

**Figure 1:**
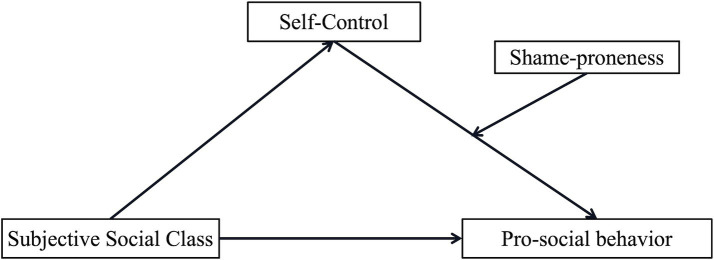
Hypothesized path model 1.

**Figure 2:**
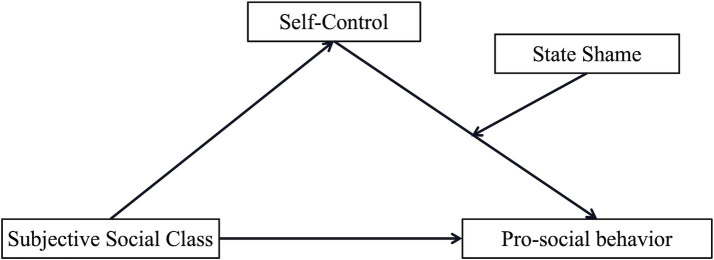
Hypothesized path model 2.

In summary, this study explores the relationship between SSC and PSB and its underlying mechanisms by establishing two moderated mediation models. In Study 1, this study used a questionnaire to measure subjects’ OSC, SSC, self-control, shame-proneness, and propensity for PSB to test Model 1 (Figure 1), which examines the mediating role of self-control and the moderating role of shame-proneness. In Study 2, a between-subjects experimental design was used to manipulate subjects’ SSC and state shame to test Model 2 (Figure 2), which examines the mediating role of self-control and the moderating role of state shame.

## 3 Study 1

Using the questionnaire method, the purpose of Study 1 was to explored: the relationship between SSC and PSB, the mediated effect of self-control and the moderated effect of shame.

### 3.1 Materials and methods

#### 3.1.1 Participants

Credamo^1^ is a professional online data platform for research (Huang et al., 2023). This study recruited participants via Credamo’s professional sampling service, which utilizes random distribution methods to ensure data authenticity and representativeness. Specifically, in the online survey, Credamo randomly distributed 340 questionnaires across 29 provinces in China to achieve broad geographic coverage and minimize sampling bias. After eliminating non-college students and participants who failed the attention test, 312 valid questionnaires were collected. The mean age was 22.71 (SD = 2.285), of which 196 were females (62.8%) and 116 were males (37.2%). Description of subjects: (1) All the participants in this study were college students in good health, with normal visual acuity or corrected visual acuity, who signed an informed consent form and received a certain amount of compensation. (2) Each experimental subject was independently recruited, and there is no case of repeated subjects.

#### 3.1.2 Procedure

Participants were recruited via an online survey platform and were instructed to complete all questionnaires in a single session in a quiet. Before beginning the survey, participants were informed about the objectives of the study, assured anonymity, and notified of their right to withdraw at any time without penalty. After reviewing the instructions and providing informed consent by checking the “Agree” box, participants commenced the survey. Participants first completed the Basic Information Questionnaire, followed sequentially by the OSC Scale, SSC Scale, Self-Control Scale, Shame Experience Scale, and PSB Scale. Standardized instructions were provided before each scale (e.g., “Please respond as honestly as possible; there are no right or wrong answers”). On average, participants required approximately 10–15 min to complete all survey sections. This procedure ensured that all measures were administered under standardized conditions and that participants clearly understood the nature and duration of the study.

#### 3.1.3 Measures

##### 3.1.3.1 SSC

**Figure 3:**
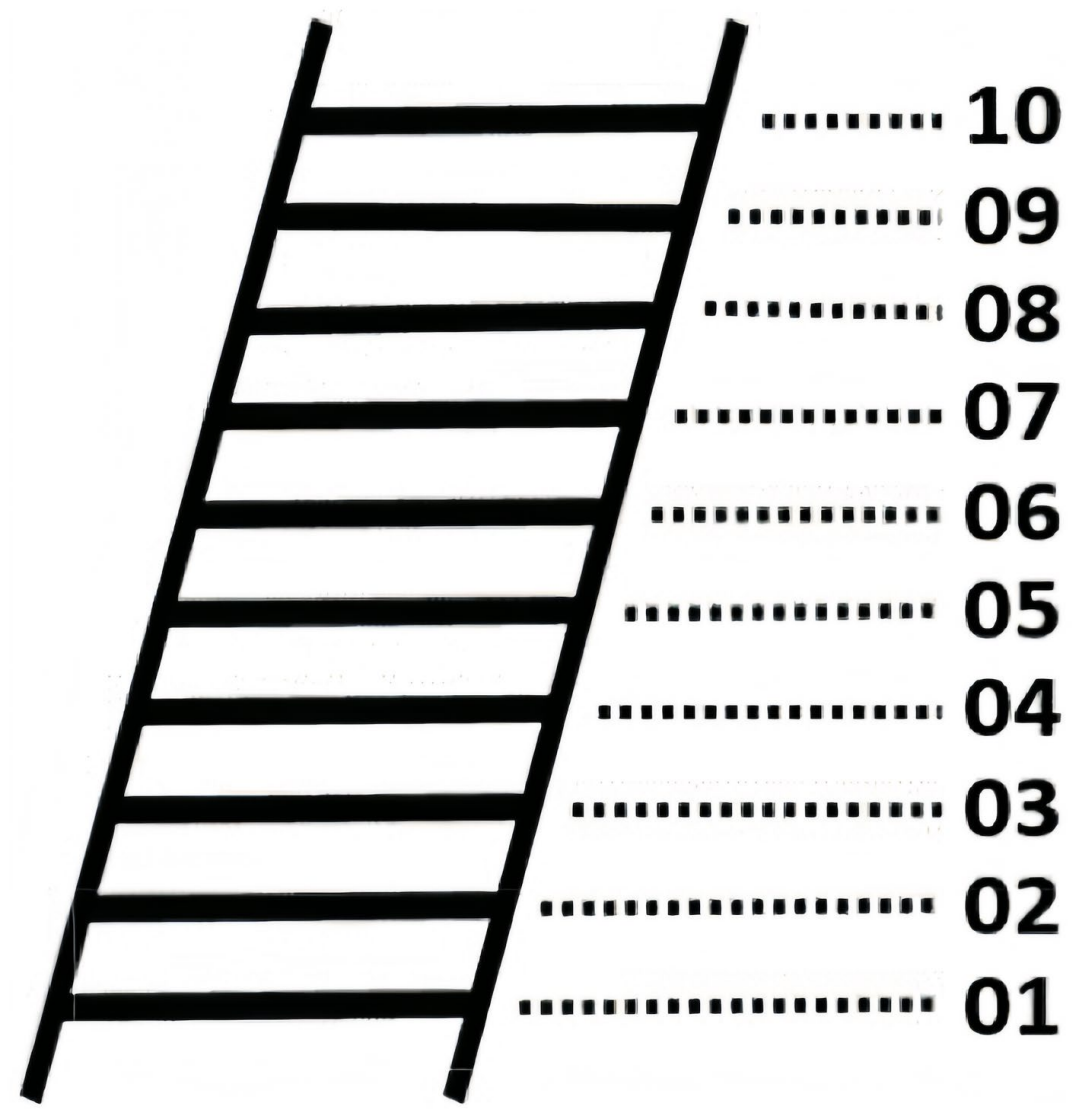
Subjective social status ladder.

The Macarthur Scale of SSC was used (Adler et al., 2000). This scale is widely used in measuring SSC with good reliability and validity (Manstead, 2018). During the measurement, the participants were presented with a 10-step ladder (Figure 3), and they were instructed to visualize that the ladder represents people’s status or class in society. Each rung represents a different economic income, educational level, and occupation. 01 for the lowest SSC, 10 for the highest SSC. Participants were asked to report their step score they belonged to in relation to the actual situation.

##### 3.1.3.2 Self-control

The latest Brief Self-Control Scale developed by Morean et al. (2014) and revised by Luo et al. (2021)was used. The scale uses a 5-point score, 1 representing completely inconsistent, and 5 representing entirely consistent’. BSCS has good reliability and validity in a sample of college students. So, it can be used as a measurement tool to study college students’ self-control level. The Cronbach’s coefficient was 0.903.

##### 3.1.3.3 Shame Experience Scale

The scale was developed by Qian et al. (2000). The scale uses a 4-point score (1 = never, 4 = often). Initially developed using Chinese college students as a norm group, this scale has been widely adopted in recent studies and has proven reliable and valid for measuring shame in college students (Lu et al., 2022). The Cronbach’s coefficient was 0.944.

##### 3.1.3.4 PSB tendency scale

The scale revised by Cong (2008) was used. The scale consists of 23 questions measuring prosocial tendencies from six dimensions: altruism, compliance, emotion, anonymity, openness, and urgency. The scale is assessed using a 5-point scoring system, with higher total scores indicating higher PSB tendencies. The Cronbach’s coefficient was 0.826.

##### 3.1.3.5 Control variables

Studies have demonstrated a moderate correlation between SSC and OSC (Zheng et al., 2021). Furthermore, individual’s SSC is more strongly associated with their behavior (Xie and Li, 2018). Hence, this study excluded the impact of OSC and concentrated on SSC to enhance the precision of the influence of SSC on PSB. Researchers measured the OSC of individuals and statistically controlled it in the data analysis. Given that the study participants were university students, the OSC is measured using family annual income, parents’ education level, and parents’ occupation as indicators (Kraus and Stephens, 2012). The measurement included five items:1. Family annual income level was rated from “below 10,000 yuan” to “above 640,000 yuan,” scored from 1 to 8 points. 2. Parents’ education level was rated from “barely literate or illiterate” to “postgraduate,” scored from 1 to 7 points. 3. Parents’ occupations were assigned values from 1 to 10 based on the classification of the “ten social strata” proposed in the context of Chinese culture (Fan, 2023). Referring to related studies, these five indicators were converted to standard scores and averaged (Chen et al., 2021).

### 3.2 Data analysis

A common method bias test was conducted using the Harman one-way test (Aguirre-Urreta and Hu, 2019). Then, Pearson correlation analysis examined the relationship between all variables. Finally, the moderated mediation model was tested using the process macro of SPSS (Model 14) (Hayes, 2022).

### 3.3 Results

#### 3.3.1 Common method bias test

Using the Harman single-factor test (Aguirre-Urreta and Hu, 2019), the results showed that 10 factors with eigenvalues greater than 1. The first common factor explained 28.636% of the total variance, which was less than the 40% judgment standard proposed by Podsakoff et al. (2012). The next step of analysis could be carried out.

#### 3.3.2 Descriptive statistics and correlation analysis of main research variables

As shown in Table 1, SSC, self-control and PSB were positively correlated with each other. Additionally, shame-proneness was negatively correlated with self-control. The correlation analysis also found a significant positive correlation between OSC and PSB.

**Table 1:** Descriptive statistics and Pearson correlations matrix (*n* = 312).

Variables	M	SD	1	2	3	4	5	6	7
1. SSC	5.37	1.193	1						
2. Self-control	27.449	4.99	0.281***	1					
3. Shame-proneness	48.202	13.238	−0.278***	−0.637***	1				
4. PSB	82.494	10.497	0.248***	0.387***	−0.227***	1			
5. OSC	0	0.81	0.434***	0.310***	−0.324***	0.144*	1		
6. Grade	1.37	0.484	−0.112*	0.048	−0.048	0.04	−0.038	1	
7. Age	22.71	2.285	0.098	0.041	−0.004	0.139*	−0.034	−0.039	1

M, mean; SD, standard deviation. *p < 0.05, ***p < 0.001. SSC, subjective social class; OSC, objective social class; PSB, Pro-social behavior.

#### 3.3.3 Moderating effects test

The moderated mediated model testing was conducted using the Process macro of SPSS 29.0. The sample size was set at 5000 with a 95% confidence interval. The independent variable was SSC; the mediator was self-control; the dependent variable was PSB; the moderator was shame-proneness. The results were as follows (see Table 2). In Model 1, SSC significantly positively predicted self-control (*β* = 0.185, *t* = 4.74, *p* < 0.001, 95% CI [0.09, 0.21]), indicating that the higher the SSC, the higher the self-control. In model 2, the predictive effect of self-control on PSB was significant (*β* = 0.457, *t* = 6.210, *p* < 0.001, 95% CI = [0.312, 0.601]), and the effect of SSC on PSB remained significant (*β* = 0.151, *t* = 2.575, *p* < 0.05, 95% CI = [0.036, 0.266]). This confirmed self-control’s mediating role. Hypotheses 1 and 2 were verified. Further testing the moderating effect of shame, the results showed that the interaction term of self-control and shame-proneness had a significant predictive effect on PSB (*β* = −0.132, *t* = −2.795, *p* < 0.01, 95% CI = [−0.226, −0.039]). This indicated that shame-proneness moderates the relationship between self-control and PSB.

**Table 2:** The moderated mediation models of self-control and shame-proneness in the relationship between SSC and PSB (*n* = 312).

Variables	Model 1 (outcome variable: self-control)			Model 2 (outcome variable: PSB)		
	*β*	SE	*t*	*β*	SE	*t*
SSC	0.185	0.06	3.090**	0.151	0.059	2.575*
OSC	0.288	0.073	3.936***	−0.015	0 0.073	−0.201
Grade	0.079	0.054	0.635	0.047	0 0.052	0.908
Age	0.034	0.054	0.635	0.132	0.052	2.523*
Self-control				0.457	0.074	6.210***
Shame-proneness				0.025	0.068	0.369
Self-control x Shame-proneness				−0.132	0.047	−2.795**
*R* ^2^	0.129			0.207		
*F*	11.407***			11.347***		

Each model is standardized and substituted into the regression equation, the same below. M, mean; SD, standard deviation. *p < 0.05, **p < 0.01, ***p < 0.001. SSC, subjective social class; OSC, objective social class; PSB, Pro-social behavior.

Values and confidence intervals for the mediating effect of self-control between SSC and PSB at different levels of shame were shown in Table 3. The mediating effect was significant when shame-proneness scores were one standard deviation below the mean (Effect = 0.109, 95% CI = [0.031, 0.209]); the mediating effect of self-control between SSC and PSB was significant but significantly weakened (Effect = 0.060, 95% CI = [0.016, 0.122]) when the shame-proneness score was higher than a standard deviation of the average. In summary, the moderated mediation model was established (Index = −0.025, Se = 0.013, 95% CI = [−0.053, −0.002]). Shame moderated the latter part of the path of the mediation effect, which verified hypothesis 3.

**Table 3:** Moderating effect of shame-proneness on the relationship between SSC and PSB (*n* = 312).

Shame-proneness	Effect	Boot SE	BootLLCl	BootULCI
M-SD	0.109	0.046	0.031	0.209
M + SD	0.06	0.028	0.016	0.122

M, mean; SD, standard deviation. SSC, subjective social class; PSB, Pro-social behavior.

**Figure 4:**
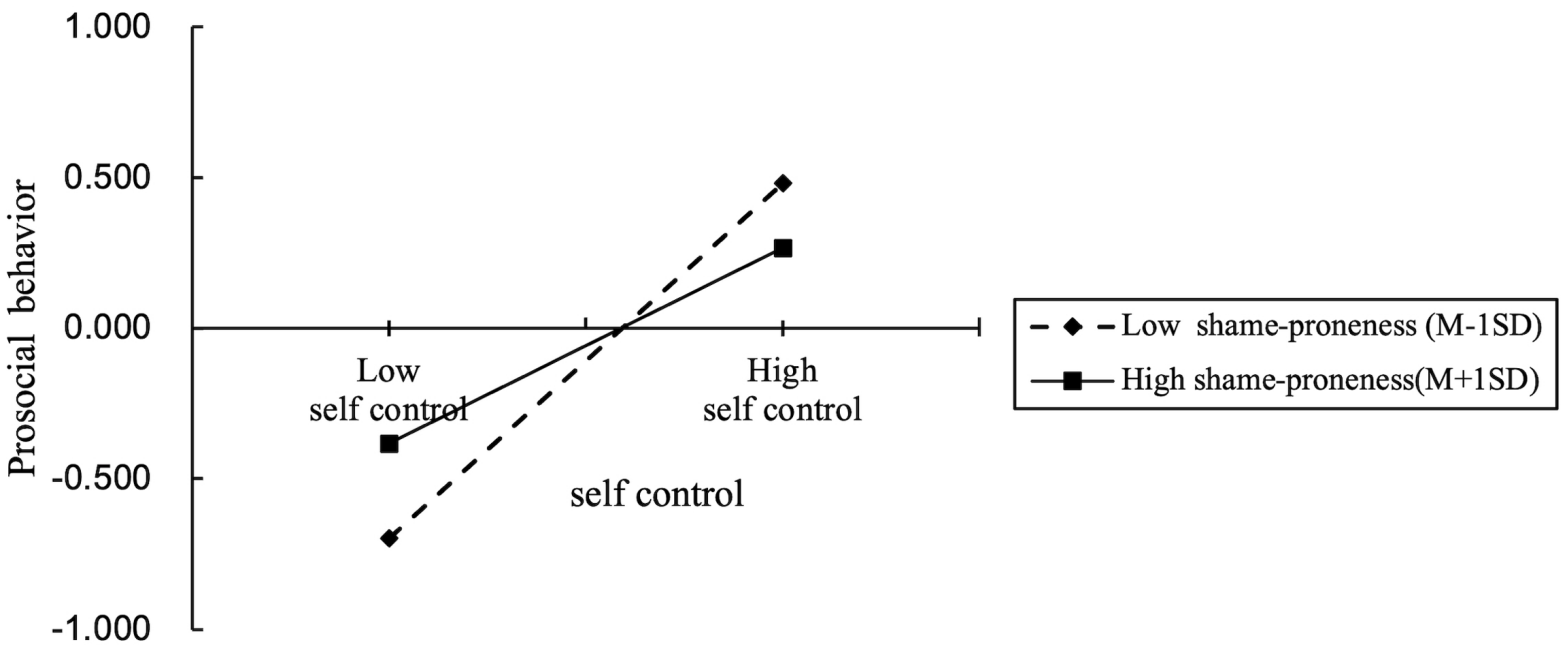
Interaction effect of the relationship between self-control and PSB at two levels of shame-proneness.

To reveal the trend of the moderating effect of shame emotion more clearly, the scores of shame emotion were divided into two groups of high and low according to one standard deviation, and the moderating effect of shame-proneness was further examined by using simple slope analysis (Figure 4). self-control positively predicted PSB when shame-proneness was high (B Simple = 0.324, *p* < 0.001), and the effect of self-control on PSB was significantly stronger when shame-proneness was low (B Simple = 0.589, *p* < 0.001). This suggested that self-control gradually increased its positive predictive effect on PSB as shame-proneness was lowered. The mediation model with moderation was established.

## 4 Study 2

Although Study 1 initially tested Hypothesis 1, 2 and 3a, the questionnaire method essentially examined the correlation between variables, could not further examine the causal relationship, and could not examine the role of state shame. Given these two problems, in study 2, researchers manipulated the SSC through experimental methods and examined the moderating effect of state shame by manipulating the individual’s shame.

### 4.1 Materials and methods

#### 4.1.1 Participants

This study used the G*power software to calculate the sample size (Faul et al., 2009), targeting medium effect sizes (Cohen’s *f* = 0.25, *α* = 0.05, 1 - *β* = 0.95) for linear multiple regression. The results indicate that a minimum of 129 participants were required. This study used the experimental method, with 257 participants from college in Zhejiang Province, China. These participants differed from those in Study 1. The experimental materials were published in the form of questionnaires on the website “Wenjuan Xing”,^2^ where participants filled out the online questionnaire to complete the experiment. The mean age of them is 19.20 (SD = 1.040), 190 (73.9%) were female. Description of subjects: (1) All participants in this study were college students, healthy, with normal vision or corrected vision, who signed an informed consent form. (2) The participants in Study 1 and Study 2 are completely distinct, with no overlap between the two groups.

#### 4.1.2 Procedure

A 2 (SSC: high vs. low) × 2 (emotion: shame vs. neutral) between-subjects experimental design was employed, with PSB as the dependent variable. Participants were recruited from eight different undergraduate classes at a university in Zhejiang Province, China. At the end of each course, the instructor displayed a QR code on the screen, allowing students to scan and access the questionnaire. Participants were informed that the survey was a psychological assessment requiring careful completion. They were provided details regarding the study’s purpose, confidentiality protocols, and their right to withdraw voluntarily. Informed consent was obtained by requiring participants to check the “Consent” box on the first page of the questionnaire. After completing the basic information, OSC and shame-proneness scale, participants were randomly assigned to one of the four experimental conditions upon providing consent.

In the initial phase of the procedure, participants were exposed to either high or low social class priming materials, followed by a brief assessment of priming effectiveness. Subsequently, participants completed the State Self-Control Scale. In the next phase, participants read either a prototypical shame-inducing scenario or a neutral event scenario to elicit the target emotion. Finally, participants engaged in a dictator game with an anonymous partner before submitting their responses. The entire procedure lasted approximately 15–20 min, with standardized instructions provided at each stage to ensure consistency in administration.

#### 4.1.3 Measures

##### 4.1.3.1 Subjective social class priming

First, using the priming paradigm designed by Piff et al. (2010), participants were exposed to a ten-rung ladder (Figure 3), to manipulate their SSC. Then, a randomly presented picture depicting the lifestyle of the highest or lowest social class was shown (Pu, 2020). The participants in the high SSC group were presented with images depicting low social class life situations, whereas the individuals in the low SSC priming group were exposed to images portraying high social class living situations. Subsequently, the participants were instructed to assess and contrast their current living circumstances with those depicted in the picture. To check if participants carefully read the materials, they were asked to answer which level of the social ladder the material presented. For the high SSC group, if the participants chose a number greater than the median 5, they failed the check; conversely, for the low SSC group, if they chose a number less than the median 5, they failed the check. Subsequently, the efficacy of the SSC priming procedure was evaluated by requesting individuals to report their present position on the ladder (Piff et al., 2010).

##### 4.1.3.2 The state self-control capacity scale

Developed by Twenge et al. (2004), this scale has proven reliable and valid (Thau and Mitchell, 2010). A seven-point Likert scale was used. It was emphasized in the instructions that participants think about their current feelings. The Cronbach’s coefficient of this scale was 0.86.

##### 4.1.3.3 Typical shame event materials

The story situation method was used to induce participants’ state shame. They were informed to envision themselves as the protagonist in the story while reading the typical shame events compiled by Gao (2006). It is worth noting that shame and guilt are similar. To ensure that shame rather than guilt was induced, guilt was also included in the assessment. After reading the typical shame events, they rated their feelings of shame and guilt on a 5-point Likert scale. According to Du (2012) ‘s scoring standard, only when the shame score was higher than four and the guilt score was lower than two did it indicate that the subject’s shame was successfully induced.

##### 4.1.3.4 Neutral emotional event materials

The neutral emotional events compiled by Du (2012) were used to induce the subjects’ neutral emotions. Many researchers have validated this material (Yang et al., 2023). After reading the neutral emotional events, participants rated their feelings of shame and guilt. If the shame and guilt scores did not exceed 2, the induction of neutral emotions was considered successful.

##### 4.1.3.5 PSB

Psychologists often use the dictator game to measure participants’ PSB (Piff et al., 2010). In this experiment, the participants were told that they would be partnered with another subject in a group and that the participants would be the ones to distribute a certain amount of money, which the other person could only accept. According to Ben-Ner et al. (2008), using actual or hypothetical money amounts in the dictator game experiment produced significantly correlated results. Both real and hypothetical money have good validity. In this experiment, the hypothetical amount of money was used, and the participants need to answer how much they are willing to give their partner and choose between “0, 20, 40, 60, 80, 100″ (Cui, 2021). The amount of money selected by the participants was the operational definition of PSB.

##### 4.1.3.6 Control variables

In study 1, researchers found a moderating effect of shame-proneness. Therefore, to better study the effect of state shame, researchers measured shame-proneness and included it as a statistical control. In addition, Similar to study 1, participants’ OSC was measured and statistically controlled in subsequent data analyses.

### 4.2 Data analysis

Firstly, we tested the common method bias. Second, we checked the correlation of variables. Third, the validity of manipulating SSC and state shame was tested. Finally, researchers examined the moderated mediation model (Hayes, 2022), the moderator was state shame.

### 4.3 Results

#### 4.3.1 Common method bias test

The results showed that eight factors were generated without rotation. The first common factor explained 32.206% of the total variance. The next step of analysis could be carried out.

#### 4.3.2 Examination of the validity of subjective social class and state shame manipulation

An independent sample t-test was performed. The results revealed that the high-class group (*N* = 126, *M* = 5.79, SD = 1.141) had significantly higher scores than the low-class group (*N* = 131, *M* = 4.11, SD = 1.26), *T* (255) = 11.17, *p* < 0.001. This confirmed the successful manipulation of the independent variable. A manipulation test of shame induction showed that shame scores (*M* = 4.30, Sd = 0.968) were significantly higher than guilt scores (*M* = 1.41, Sd = 0.823) in the shame events. The differences between the means of the shame and guilt were above 2 points, indicating that the shame event used in this study was effective in inducing shame in the subjects. In the neutral emotion event, the guilt score (*M* = 1.20, SD = 0.515) and the score of the state shame (*M* = 1.92, SD = 0.932) were below 2 points, meaning that the neutral emotion event chosen in this study was appropriate (Table 4).

**Table 4:** Examination of the validity of state shame manipulation (*n* = 257).

	Shame event (*n* = 122)	Neural event (*n* = 135)	*t*	*p*	Cohen d
Shame score	4.30 (0.968)	1.41 (0.823)	25.77***	<0.001	0.895
Guilt score	1.92 (0.932)	1.2 (0.515)			

***p < 0.001.

#### 4.3.3 Descriptive statistics and Pearson correlations analysis of main research variable

As shown in Table 5, SSC, self-control and PSB were positively correlated with each other. Additionally, shame-proneness was correlated with PSB.

**Table 5:** Descriptive statistics and Pearson correlations matrix (*n* = 257).

Variables	M	SD	1	2	3	4	5	6	7	8	9
1. SSC	4.94	1.467	1								
2. Self-control	42.377	11.384	0.449^***^	1							
3. State shame	2.78	1.695	−0.046	−0.05	1						
4. PSB	46.77	15.789	0.383^***^	0.319^***^	0.289^***^	1					
5. Shame -proneness	52.257	14.239	−0.112	−0.293^***^	0.161^**^	−0.018	1				
6. OSC	0	0.771	0.339^***^	0.135^*^	−0.191^**^	0.091	−0.245^***^	1			
7. Guilt	1.54	0.824	0.018	0.005	0.496^***^	0.036	0.077	−0.142^*^	1		
8. Grade	1.74	0.44	−0.031	0.091	0.081	−0.026	0.041	−0.130^*^	0.056	1	
9. Age	19.2	1.04	0.011	0.075	−0.046	0.151^*^	−0.048	−0.037	−0.048	−0.262^***^	1

M, mean; SD, standard deviation. *p < 0.05, **p < 0.01, ***p < 0.001. SSC, subjective social class; OSC, objective social class; PSB, Pro-social behavior.

#### 4.3.4 Moderated mediation effects test

Model 14 (Bootstrap sampling is 5,000) in the Process macro program was used. Variables were standardized before formal data processing. Age, gender, shame-proneness, and OSC were control variables. The test results were as follows (Table 6). In Model 1, SSC significantly positively predicted self-control (*β* =0.443, t = 7.767, *p* < 0.001, 95%CI = [0.331, 0.555]). After adding the mediator variable self-control, in Model 2, self-control significantly positively predicted PSB (*β* =0.211, *t* = 3.406, *p* < 0.001, 95%CI = [0.089, 0.332]), and SSC still significantly predicted PSB (*β* =0.313, *t* = 5.056, *p* < 0.05, 95%CI = [0.191, 0.435]). This indicated that self-control mediates the relationship between SSC and PSB. hypotheses 1 and 2 were verified. Further testing of the moderating effect of shame showed that the interaction term between self-control and state shame significantly predicted PSB (*B* = −0.125, *t* = −2.437, *p* < 0.05, 95%CI = [−0.227, −0.024]). This indicated that state shame moderates the relationship between self-control and PSB.

**Table 6:** The moderated mediation models of state shame in the relationship between self-control and PSB (*n* = 257).

Variables	Model 1 outcome variable: self-control			Model 2 outcome variable: PSB		
	*β*	SE	*t*	*β*	SE	*t*
SSC	0.443	0.057	7.767***	0.313	0.062	5.056***
OSC	−0.074	0.077	−0.963	0.058	0.076	0.761
Guilt	0.006	0.054	0.103	−0.155	0.06	−2.579*
Shame proneness	−0.259	0.055	−4.69***	0.038	0.057	0.671
Grade	0.131	0.056	2.345*	−0.003	0.056	−0.047
Age	0.091	0.056	1.633	0.139	0.055	2.541*
Self-control				0.211	0.062	3.406***
State shame				0.401	0.061	6.569***
Self-control X State shame				−0.125	0.051	−2.437*
*R* ^2^	0.286			0.33		
*F*	16.693***			13.543***		

Each model is standardized and substituted into the regression equation, the same below. *p < 0.05,***p < 0.001. SSC, subjective social class; OSC, objective social class; PSB, Pro-social behavior.

The mediating effect and confidence interval of self-control between SSC and PSB in different shame emotions were shown in Table 7. When the state shame score is lower than a standard deviation of the average, the mediating effect of self-control is significant (Effect = 0.336,95% CI = [0.172,0.500]); when the state shame score was higher than a standard deviation of the average, the mediating effect was not significant (Effect = 0.085, 95% CI = [−0.067, 0.238]). In summary, the moderated mediation model was established (Index = −0.056, SE = 0.023, 95% CI = [−0.103, −0.014]), and state shame moderated the second half of the mediating effect, which verified hypothesis 3.

**Table 7:** Moderating effect of state shame on the relationship between SSC and PSB (*n* = 257).

Shame	Effect	Boot SE	BootLLCl	BootULCI
M-SD	0.149	0.044	0.068	0.238
M + SD	0.038	0.03	−0.022	0.097

M, mean; SD, standard deviation; SSC, subjective social class; PSB, Pro-social behavior.

**Figure 5:**
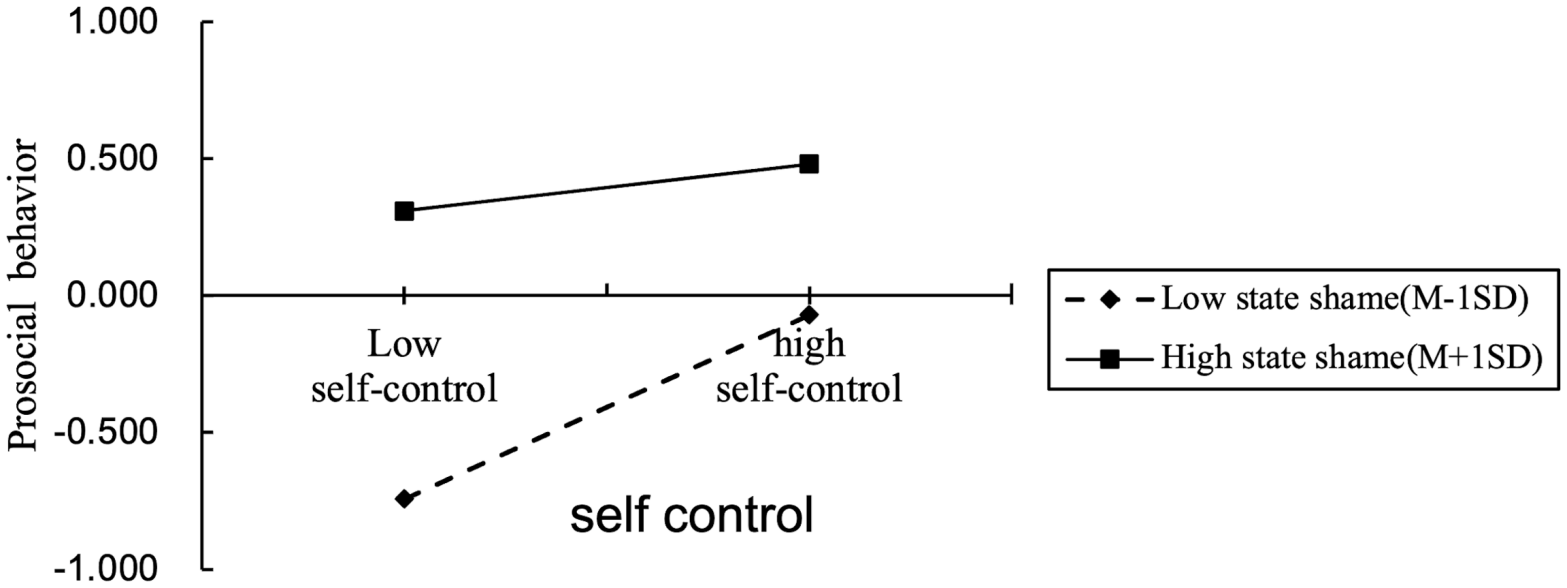
Interaction effect of the relationship between self-control and PSB at two levels of state shame.

To reveal the trend of the effect of shame emotion more clearly, the scores of shame emotion were divided into two groups of high and low according to one standard deviation of positive and negative. The moderating effect of state shame between self-control and PSB was further examined by using simple slope analysis. As shown in Figure 5, when state shame was low, self-control had a positive predictive effect on PSB (B Simple = 0.336, *p* < 0.001); when state shame was high, the predictive effect of self-control on PSB was not significant (B Simple = 0.085, *p* > 0.05). This indicated that as state shame decreases, the positive predictive effect of self-control on PSB gradually strengthens. The moderated mediation model was established.

## 5 General discussion

### 5.1 SSC and PSB

This study found that SSC was positively associated with PSB. This finding is consistent with previous research (Kraus and Callaghan, 2016). The Cost Consumption Theory posits that PSB involve certain costs—such as time, money, or social capital—and those higher perceived costs reduce individuals’ willingness to help (Korndörfer et al., 2015). Since individuals with lower SSC have limited access to resources, the relative cost of helping is greater, thereby decreasing their likelihood of engaging in PSB. In contrast, individuals with higher SSC have greater resources and encounter fewer social constraints, which enables them to better bear the costs assisting others (Li et al., 2024). Thus, our findings align with this theoretical perspective and provide novel empirical evidence for understanding how SSC influences OSB.

Previous studies have demonstrated that an individual’s SSC is more strongly correlated with their behavior than OSC (Xie and Li, 2018). For example, SSC is a stronger predictor of PSB than OSC (Manstead, 2018), indicating that subjective perceptions of social status play a more prominent role in shaping prosocial tendencies. Our results further indicate that PSB is more likely exhibited by individuals with a high SSC, regardless of their OSC. Therefore, enhancing individuals’ perceptions of their SSC may be an effective strategy to promote PSB among college students.

### 5.2 The mediating role of self-control

This study found that SSC indirectly promotes PSB through self-control. The findings support the following theoretical frameworks. First, Scarcity Theory posits that individuals with limited resources undergo psychological depletion, including a reduction in self-control (Mullainathan and Shafir, 2013). Individuals facing scarcity allocate cognitive resources primarily to immediate needs, thereby reducing self-regulatory capacities and ultimately impairing self-control (Sun et al., 2024). Our study further corroborates this finding, demonstrating that individuals with low SSC possess fewer self-control resources and are thus more inclined toward egoistic behaviors rather than PSB in scarcity contexts. Second, the self-control process model of altruistic behavior suggests that engaging in PSB necessitates self-control resources to suppress egoistic tendencies (Fei et al., 2016). In other words, self-control functions as a protective factor that facilitates PSB engagement (Zhang et al., 2021). Joosten et al. (2015) further demonstrated that when individuals’ self-control resources are depleted, their moral decision-making abilities and PSB levels decline significantly.

This study additionally presents novel findings. Unlike previous studies that primarily examined the direct impact of SSC on PSB, this study further explores the mediating role of self-control in this process. The mediating pathway identified in this study aligns with The Cost Consumption Theory (Korndörfer et al., 2015). Engaging in PSB necessitates resource expenditure (self-control). Individuals with high SSC possess greater self-control resources, enabling them to override self-serving impulses and engage in PSB. Conversely, individuals with low SSC have fewer self-control resources and lack the necessary resources to engage in PSB, making them less likely to do so. In summary, this study confirms the mediating role of self-control and provides empirical support for Hypothesis 2.

### 5.3 The moderating role of shame

Studies 1 and 2 revealed that both trait shame (shame-proneness) and state shame function as negative moderators in the relationship between self-control and PSB. However, the mechanisms through which these two forms of shame influence PSB differ. In Study 1, shame-proneness reduced PSB among individuals with high SSC, whereas in Study 2, state shame enhanced PSB among low-SSC individuals. These seemingly contradictory patterns align with previous research, which suggests that discrete, momentary shame (state shame) is more likely to trigger approach-oriented or restorative behaviors, whereas chronic, internalized shame (shame-proneness) tends to induce withdrawal or avoidant responses (Hao and Cui, 2022). According to Hooge ‘s motivation theory, individuals who encounter shame will have the motivation to restore and protect the positive self to deal with the threatened self. These two motivations determine whether subsequent behavior will be approach- or avoidance-oriented. On the one hand, shame activates approach-oriented behaviors aimed at restoring the threatened self; on the other hand, it triggers avoidance behaviors to protect the self from further harm when individuals perceive recovery as unattainable (De Hooge et al., 2010). Meanwhile, the Resource-Allocation Model of Self-Control posits that individuals allocate self-control resources based on their behavioral motivations to achieve their goals (Beedie and Lane, 2012). Thus, the influence of self-control on PSB is moderated by shame.

Specifically, when shame is transient (state shame) and individuals perceive that they can restore their threatened self-concept, a restorative motivation is activated (de Hooge et al., 2018). This restorative motivation compels individuals to enhance their self-image through subsequent behaviors, necessitating the mobilization of additional self-control resources (Yang et al., 2023). Thus, state shame enhances self-control (Zhong et al., 2010), which in turn serves as a protective factor for PSB (Fei et al., 2016). Therefore, state shame may increase PSB among individuals with low SSC. Conversely, when shame becomes a chronic, stable disposition (shame-proneness), individuals are more likely to perceive self-image restoration as unattainable or excessively costly (Hao and Cui, 2022). This belief fosters a protective motivation, which depletes self-control resources by prioritizing self-protection and minimizing further exposure to negative evaluation (Tangney et al., 2007). Consequently, individuals with high shame-proneness are less inclined to engage in prosocial actions (Wang and Li, 2020), as a substantial portion of their cognitive and emotional energy is allocated to shielding themselves from further judgment. Thus, while both forms of shame negatively moderate the link between self-control and PSB, they do so via different mechanisms. State shame, being momentary and context-specific, can spark a restorative drive that temporarily heightens self-control and increases prosocial tendencies among individuals of lower SSC. In contrast, shame-proneness, being chronic and tied to a stable self-view, engenders a protective drive that drains self-control resources and suppresses PSB among individuals of higher SSC.

### 5.4 Implications

From a practical perspective, this study underscores the mediating role of self-control, offering a foundation for interventions designed to enhance self-control and promote PSB among college students. For instance, educators can implement interventions such as mindfulness training, structured reflection sessions, and self-monitoring activities to enhance self-control and encourage PSB. Moreover, this study reveals that shame operates as a double-edged sword—chronic shame-proneness reduces PSB among individuals from higher social classes, whereas transient state shame enhances it among those from lower social classes. Therefore, educational institutions, families, and policymakers should address the issue of shame with ethical sensitivity. For instance, moral education programs should help students develop an appropriate perspective on shame, recognizing that while moderate shame may facilitate moral behavior to some extent, excessive humiliation can be ethically problematic and potentially harmful. Educational institutions should reinforce moral education among college students, fostering the development of sound moral values and ethical awareness. Educators should adopt restorative approaches (e.g., peer mediation or guided discussions) instead of punitive measures. This balanced approach can help cultivate a morally conscious educational environment and foster social harmony.

## 6 Limitations and future research directions

This study has limitations, and subsequent research can take these perspectives to further advance the findings of this study. First, the participants were college students. The reason for this selection is the fact that social class is inherent to them, so it is easier to make a causal judgment on the influence of their class attributes on their behaviors. However, the college student population may not fully understand their class attributes, and thus caution is warranted when generalizing the findings. Furthermore, Study 2 had a disproportionate number of female participants. Although neither previous research nor the present study found significant gender differences in PSB (Zheng et al., 2021), future research could enhance sample representativeness.

Several issues remain to be explored in future research. First, this study focused solely on self-control. A more comprehensive exploration of the underlying psychological mechanisms could provide additional empirical support for promoting PSB among college students. Second, PSB in this study were measured using the dictator game paradigm. Future research could explore the impact of SSC on other forms of PSB (e.g., cooperation and donation) and investigate their underlying psychological mechanisms. Third, the research methods employed were relatively conventional. Future research could utilize big data and qualitative methods to investigate related questions and provide a more in-depth exploration of the conclusions.

## 7 Conclusion

The higher the level of SSC among college students, the higher the level of PSB. This behavior occurred in part through self-control. State shame and shame-proneness negatively moderated the relationship between self-control and PSB; the lower the level of shame, the greater the effect of self-control on PSB.

## Data Availability

The original contributions presented in the study are included in the article/supplementary material, further inquiries can be directed to the corresponding author.

## Footnotes

^1^ https://www.credamo.com/#/

^2^ https://www.wjx.cn
